# Risk perception of health problems among travelers visiting a travel clinic in Bangkok, Thailand

**DOI:** 10.1186/s40794-020-00108-0

**Published:** 2020-05-20

**Authors:** Thanyapat Hiranrusme, Watcharapong Piyaphanee, Jaranit Kaewkungwal, Udomsak Silachamroon, Wattana Leowattana, Lapakorn Chatapat, Wasin Matsee

**Affiliations:** 1 Department of Clinical Tropical Medicine, Faculty of Tropical Medicine, Mahidol University, 420/6 Ratchawithi Road, Bangkok, 10400 Thailand; 2 Department of Tropical Hygiene, Faculty of Tropical Medicine, Mahidol University, Bangkok, Thailand

**Keywords:** Risk perception, Health problems, Travelers, Pre-travel consultation, Travel health risk

## Abstract

**Background:**

Effective pre-travel consultations cannot be achieved only through individual risk assessment and advice on vaccinations and chemoprophylaxis. Travelers’ perceptions of the risk of health problems represent another key factor in successful risk communication and co-operation with pre-travel advice. The objective of this study was to determine perception of travel-related health risks among Thais and westerners visiting the Thai Travel Clinic for consultation before visiting developing countries.

**Methods:**

A novel pictorial scale questionnaire-based study was conducted with both Thai and western travelers who visited the Thai Travel Clinic for pre-travel consultation. All participants were approached before and after completing the consultation, and were asked about their demographic data and perceptions of travel-related health risk. The perceptions of risk before and after consultation were compared using the McNemar test, and were also compared with the actual estimated risk.

**Results:**

During May to November 2019, 594 travelers (330 Thais and 264 Westerners) were enrolled and completed the pictorial scale questionnaires. Most Thai travelers visited Africa/South America (63%), and 20% had previously received counseling. Westerners were mostly backpackers (37.5%), traveling for > 30 days (71.6%), while 43.6% had previously received counseling. Overall, the westerners (*n* = 264) changed their risk perceptions slightly after counseling in contrast with the Thais. The change in perception of most health problems was observed statistically significant (*p*-value < 0.05) after receiving pre-travel consultation among both groups of travelers. Risk perception among western travelers after consultation compared with estimated actual risk showed accurate risk perception toward most of health problems especially in travelers who had previously received counseling in ones’ home countries.

**Conclusions:**

Risk perception of health problems plays an important role in successful risk communication and their response to pre-travel advices. Differences in risk perceptions were evident between the two groups. Therefore, this highlight the importance of obtaining pre-travel advice in one’s home country before travelling. Raised awareness of the risks should be emphasized during consultations for underestimated health risks, especially for rabid animal exposure and sexually transmitted diseases.

## Background

International travel has continued growing annually with the rapid evolution of global travel patterns [1, 2]. Changes in global travel patterns have increased the complexity of travel medicine approaches. Travel medicine has recently emerged as a medical specialty in Thailand with a recognized national board-certified residency training program [3]. Pre-travel consultation, with individual risk assessment, aims to address travelers’ risks related to their itineraries and minimize those risks by educating travelers and providing appropriate vaccination(s) and chemoprophylaxis [4]. The estimated risk numbers proposed by Steffen R [5, 6]. are usually addressed during pre-travel advice to communicate risk before travel to developing countries. Previous report in Thai travelers has demonstrated that lack of awareness and poor knowledge in malaria risk in Sub-Saharan Africa could result in fatal outcome [7]. So, there is an urgent need to raise awareness of various health risk during pre-travel consultation. Nevertheless, travelers’ perceptions of risk play an important role in successful risk communication and their response to pre-travel advice [8, 9]. Currently, our knowledge of risk perceptions is limited because of difficulties with evaluation and the interpretation of results [10]. We currently lack an effective method for assessing perception of risk, especially one immediately comparable with standard estimated actual risk scales [5, 6]. Therefore, in this study, we used a new measurement method to assess travelers’ risk perceptions. By using this tool, we aimed to determine risk perceptions of health problems among travelers before and after pre-travel counseling.

## Methods

### Study population and samples

**Fig. 1:**
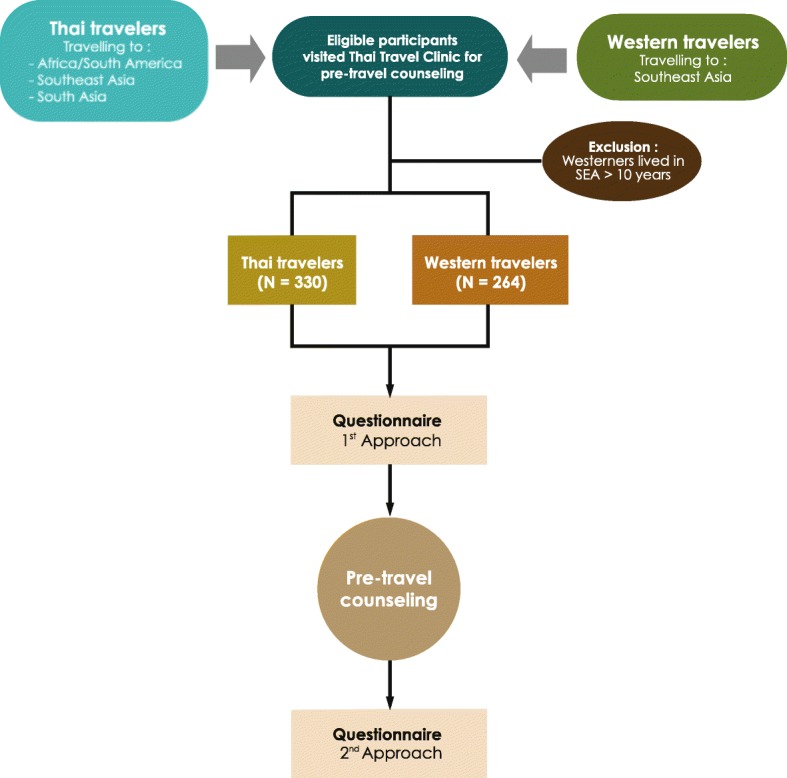
Flowchart of study enrollment and methodology

This was a pictorial scale questionnaire-based study. Data were collected from both Thai and western travelers who sought pre-travel counseling at the Thai Travel Clinic, Hospital for Tropical Diseases, Bangkok, Thailand during the period May to November 2019. Eligible participants were 1) Thai travelers who traveled to any countries in Africa/South America, Southeast Asia or South Asia; 2) western travelers to Southeast Asia, defined as travelers who came from North America, Europe, Australia, and New Zealand, who could understand and were willing to complete the English language questionnaire; 3) aged 18 years or older (both male and female); and 4) first visit to the Thai Travel Clinic. Foreign expatriates living in Thailand were excluded. The investigating team invited all eligible participants to enroll in the study. Written informed consent was obtained before enrollment. After enrollment, all participants were asked to complete the questionnaire before and after pre-travel consultation. All participants followed the regular process of the clinic (visited the physician, received pre-travel advice). Pre-travel counseling was provided by travel-medicine physicians with at least 3 years’ experience in the travel clinic. This process generally took 15–20 min per participant. A flowchart of study enrollment and the methodology used is shown in Fig. 1.

The study protocol and questionnaire were reviewed and approved by the Ethics Committee of the Faculty of Tropical Medicine, Mahidol University. Study participants were recruited by convenience-sampling technique from travelers visiting the Thai Travel Clinic during the data-collection period.

### Measurement of perception

**Fig. 2:**
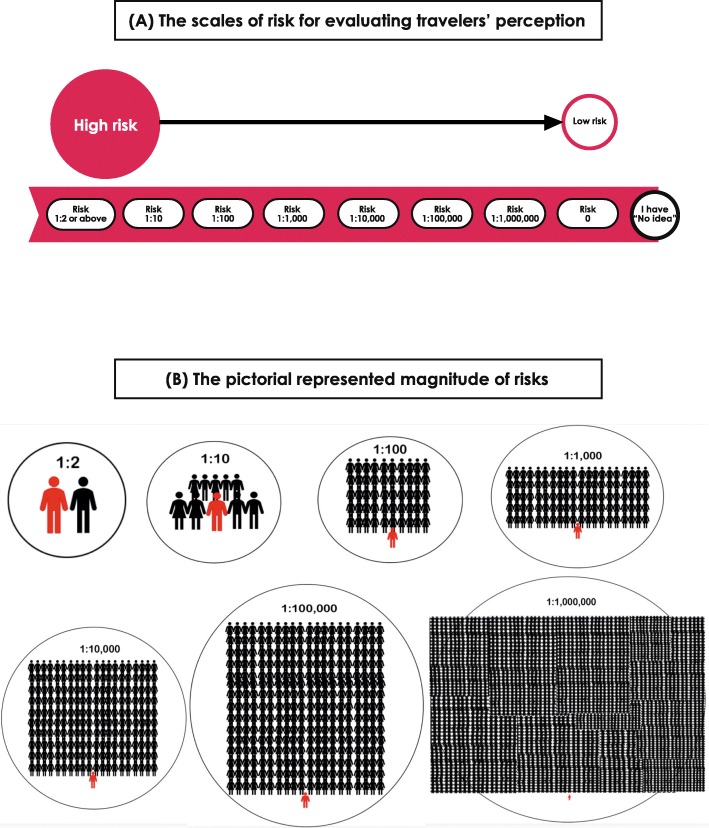
Pictorial scales used for risk evaluation

The study questionnaire had two versions, Thai and English and comprised of two parts—demographic information and a pictorial scale to assess risk perception. The perceptions of travelers were evaluated for 14 travel-related infectious and non-infectious health problems: malaria, Japanese encephalitis, hepatitis A, travelers’ diarrhea, rabid animal exposure, influenza, sexually transmitted diseases, traffic accident, physical/sexual assault, robbery, vaccine adverse events, psychiatric problems, sunburn, and natural disaster. A numerical scale with a pictorial diagram modified from PRISM [11] was attached to the questionnaire to assist participants visualize the magnitude of risk (Fig. 2). The scale for estimated actual risk proposed by Steffen R [5, 6] was used to evaluate risk perception of travelers. The frequency of the scale ranged between zero risk (lowest risk), and risk of 1 in 2 and above (highest risk). Travelers were invited to select one best answer on the scales of risk against the aforementioned 14 health problems. An optional answer—“I have no idea”—was available for participants who had no perception of the risk.

Before ethical approval and official use in this study, the questionnaire was validated for comprehensibility by a trial with 30 random travelers.

### Statistical analysis

All statistical analyses were performed using SPSS version 18 software for Windows (SPSS Inc., Chicago, IL, USA). Continuous data were presented as median with range (for non-normally distributed data). Categorical data were presented as numbers and percentage. The McNemar test was used to compare 8 variables of travelers’ perceptions on each risk between before and after consultation for all 14 travel-related health risk. A two-sided *p* value < 0.05 was considered statistically significant. The mode for each risk perception of a health problem was selected and plotted on the scale similarly with estimated actual risk by Steffen R [5, 6]. The risk perception after consultation was compared with actual risk for each travel health problem.

## Results

### Demographics and travel characteristics

All 594 participants, 330 Thais and 264 westerners, completed the questionnaires. Of the 330 Thai travelers, 37% were in the age group 30–39 years. The highest level of education was predominantly bachelor’s degree or above (*n* = 289, 87.6%). The main purpose of travel was tourism (*n* = 205, 62.1%) with a duration of 8–15 days (*n* = 149, 45.2%). The main intended destinations were Africa & South America (*n* = 208, 63%), followed by South Asia (*n* = 69, 20.9%), and Southeast Asia (*n* = 53, 16.1%).

Of 264 western travelers, 53.8% were in the age group 18–29 years. Most were European nationality (*n* = 139, 52.65%), followed by North America (*n* = 106, 40.15%) and Australia/New Zealand (*n* = 19, 7.19%). The highest level of education was bachelor’s degree or above (*n* = 191, 72.4%). Most were backpackers (*n* = 99, 37.5%) and planned to travel > 30 days (*n* = 189, 71.6%) in Southeast Asia; 43.6% (*n* = 115) had visited a travel clinic for pre-travel counseling before departure. The demographic data and characteristics of the participants are presented in Table 1.

**Table 1:** Demographic characteristics of study participants

Characteristics	Thais (*N* = 330)	Westerns (*N* = 264)
**Sex (%)**		
Male	149 (45.2%)	163 (61.7%)
Female	181 (54.8%)	101 (38.3%)
**Age (%)**		
18–29	86 (26.1%)	142 (53.8%)
30–39	122 (37%)	57 (21.6%)
40–49	88 (26.7%)	36 (13.6%)
50–59	25 (7.6%)	14 (5.3%)
60 and above	9 (2.7%)	15 (5.7%)
**Education (%)**		
Below High school	7 (2.1%)	4 (1.5%)
High school/Diploma	34 (10.3%)	69 (26.1%)
Bachelor’s degree	160 (48.5%)	109 (41.3%)
Above Bachelor	129 (39.1%)	82 (31.1%)
**Country of origin (%)**		
Europe	–	139 (52.65%)
North America	–	106 (40.15%)
Australia/ New Zealand	–	19 (7.19%)
**Length of stay (%)**		
1–7 days	24 (7.3%)	6 (2.3%)
8–15 days	149 (45.2%)	16 (6.1%)
16–30 days	51 (15.5%)	53 (20.1%)
> 30 days	106 (32.1%)	189 (71.6%)
Median + SD	15 + 232.3	58 + 172
**Purpose of travel (%)**		
Tourism	205 (62.1%)	208 (78.8%)
Business	102 (30.9%)	25 (9.5%)
VFR	2 (0.6%)	9 (3.4%)
Volunteer/Mission	3 (0.9%)	4 (1.5%)
Study/Lecture/Research	18 (5.5%)	18 (6.8%)
**Characteristic of travel (%)**		
Travel alone with hotel stay	91 (27.6%)	82 (31.1%)
Travel with friends & relatives	130 (39.4%)	80 (30.3%)
Group tour	63 (19.1%)	3 (1.1%)
Backpacker	46 (13.9%)	99 (37.5%)
**Activity (%)**		
Hiking	73 (22.1%)	95 (36%)
Spelunking	7 (2.1%)	5 (1.9%)
Diving	16 (4.8%)	25 (9.5%)
Safari	18 (5.5%)	1 (0.4%)
No adventure activity	216 (65.5%)	138 (52.3%)
**Destination (%)**		
Southeast Asia	53 (16.1%)	264 (100%)
Africa/South America	208 (63%)	0 (0%)
South Asia	69 (20.9%)	0 (0%)
**Trip experience (%)**		
yes	93 (28.2%)	164 (62.1%)
no	237 (71.8%)	100 (37.9%)
**Visited before travel (day)**		
Mean + SD	28.8 + 33.2	
**Previous counseling (%)**	66 (20%)	115 (43.6%)
**Underlying diseases (%)**	57 (17.3%)	48 (18.2%)
**Medication used (%)**	48 (14.5%)	67 (25.4%)

### Risk perceptions before and after pre-travel counseling

**Fig. 3:**
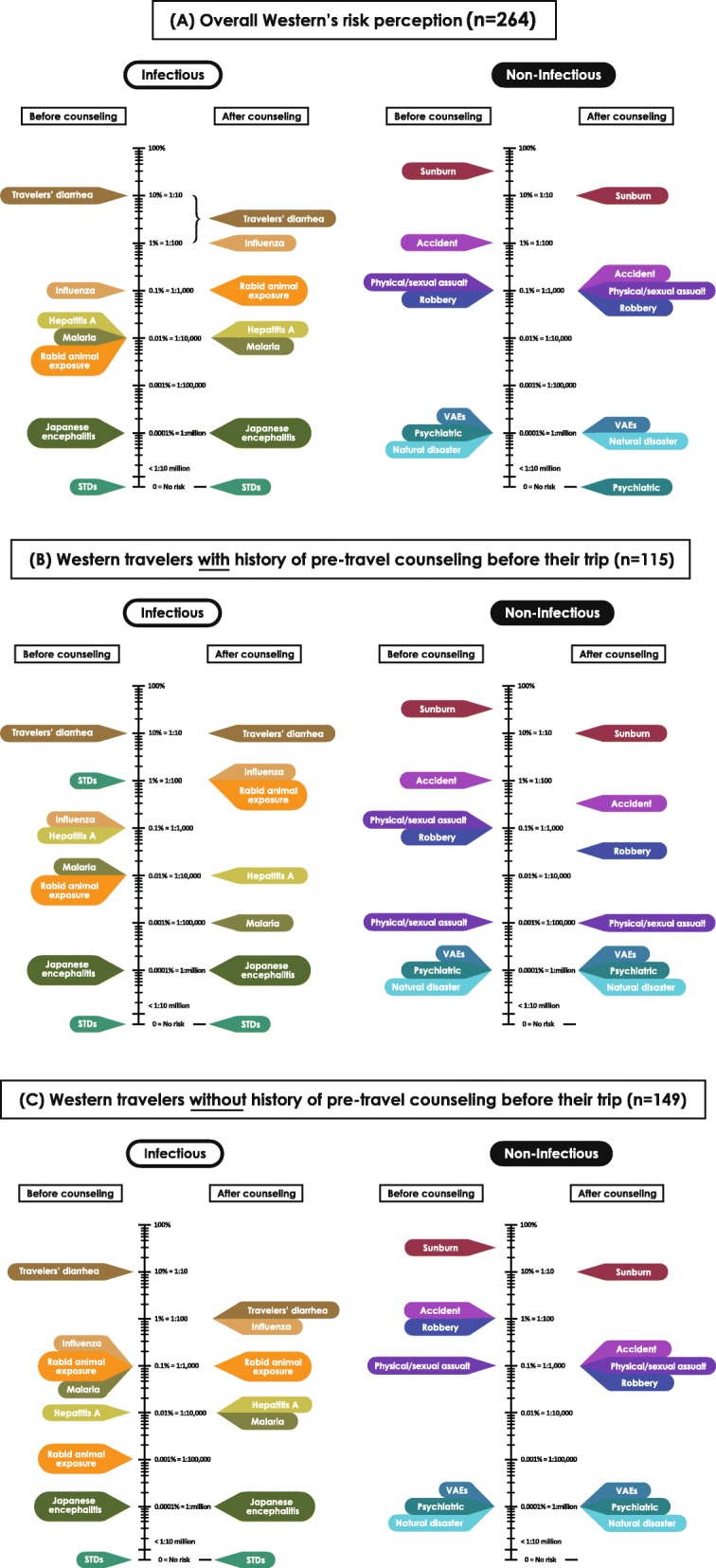
Westerners’ perceptions of risk before and after counseling

Before the pre-travel advice, the westerners (*n* = 264) perceived the risk of malaria and rabid animal exposure at 0.01% (1 in 10,000), while STDs were perceived as zero risk. After pre-travel advice, the risk perception for malaria (0.01%) and STDs (zero) remained unchanged, whereas the risk perception of rabid animal exposure increased to 0.1% (1 in 1000). Westerners’ perceptions of risk against all 14 health problems are shown in Fig. 3, where details of perceptions before and after counseling were classified into 3 groups: (A) overall perception (*N* = 264), (B) with history of pre-travel counseling before their trip (*N* = 115), and (C) without history of pre-travel counseling before their trip (*N* = 149).

**Fig. 4:**
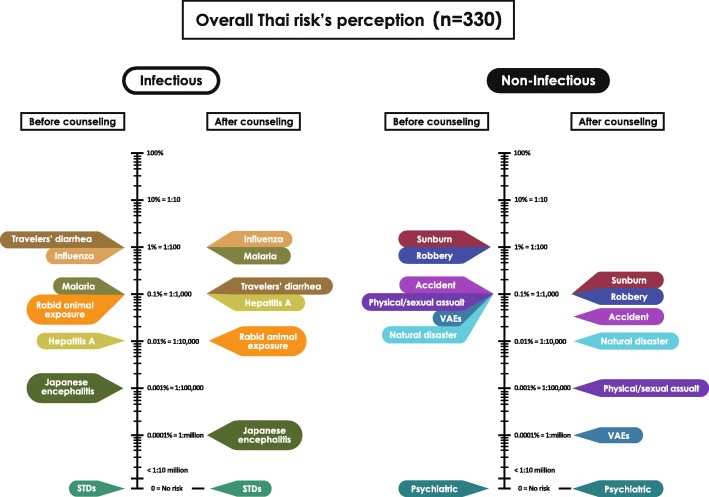
Thais’ perceptions of risk before and after counseling

For the Thai participants, before counseling, the overall perception (*n* = 330) of risk for malaria and rabid animal exposure were 0.1% (1 in 1000), while the risk of STDs was perceived as zero. After counseling, a change in risk perception was observed for malaria (1%, or 1 in 100, *p* = 0.000), and rabid animal exposure (0.01%, or 1 in 10,000, *p* = 0.000), whereas, the perceived risk of STDs remained unchanged, at zero (*p* = 0.017). The overall result for the Thais’ perceptions of risk for all 14 travel-related health problems, comparing before and after pre-travel counseling, is shown in Fig. 4.

The reduction of “I have no idea” response was observed significantly after post-travel consultation toward all health problems. The percentage of travelers who response “I have no idea” on pre- and post-travel counseling was described in Table 2. The distribution of answers among travelers demonstrating risk perception of each health problems were illustrated in funnel plots. (see detail in Additional file 1).

**Table 2:** Percentage of response “I have no idea” among travelers in comparison of pre- and post-counseling

Health problems	Thai travelers			Western travelers		
	Pre-counseling (%)	Post-counseling (%)	*p*-value	Pre-counseling (%)	Post-counseling (%)	*p*-value
Malaria	23.9	7.9	0.000	14.0	1.1	0.003
Japanese encephalitis	38.8	16.4	0.000	20.5	2.3	0.000
Hepatitis A	30.0	9.7	0.000	20.1	3.4	0.001
Travelers’ diarrhea	13.9	3.3	0.009	9.5	1.9	0.009
Influenza	14.8	6.4	0.006	23.1	6.8	0.000
Rabid animal exposure	21.5	8.2	0.000	12.9	2.3	0.001
STDs	14.8	6.1	0.017	20.5	5.7	0.000
Accident	12.1	3.9	0.003	11.7	2.7	0.015
Physical/sexual assault	14.5	4.5	0.004	12.9	4.2	0.027
Robbery	11.2	3.0	0.001	12.1	4.2	0.054
Vaccine adverse events	24.5	5.2	0.000	24.2	4.9	0.000
Psychiatric problems	16.7	4.8	0.000	21.2	6.8	0.005
Sunburn	12.4	3.6	0.000	8.7	1.9	0.017
Natural disaster	22.1	8.2	0.000	18.2	4.9	0.000

Subgroups analysis was performed among western travelers, which showed no statistically significant of the change in perception for age and gender. However, the European was the only group that found a statistically significant change in perception after consultation toward the risk for rabid animal exposure (*p* = 0.018) and STDs (*p* = 0.007). The perception on STDs after counseling was found remarkably risen from zero to 0.01% (1:10,000) (see detail in Additional file 2).

### Comparison of risk perception and estimated actual risk

**Fig. 5:**
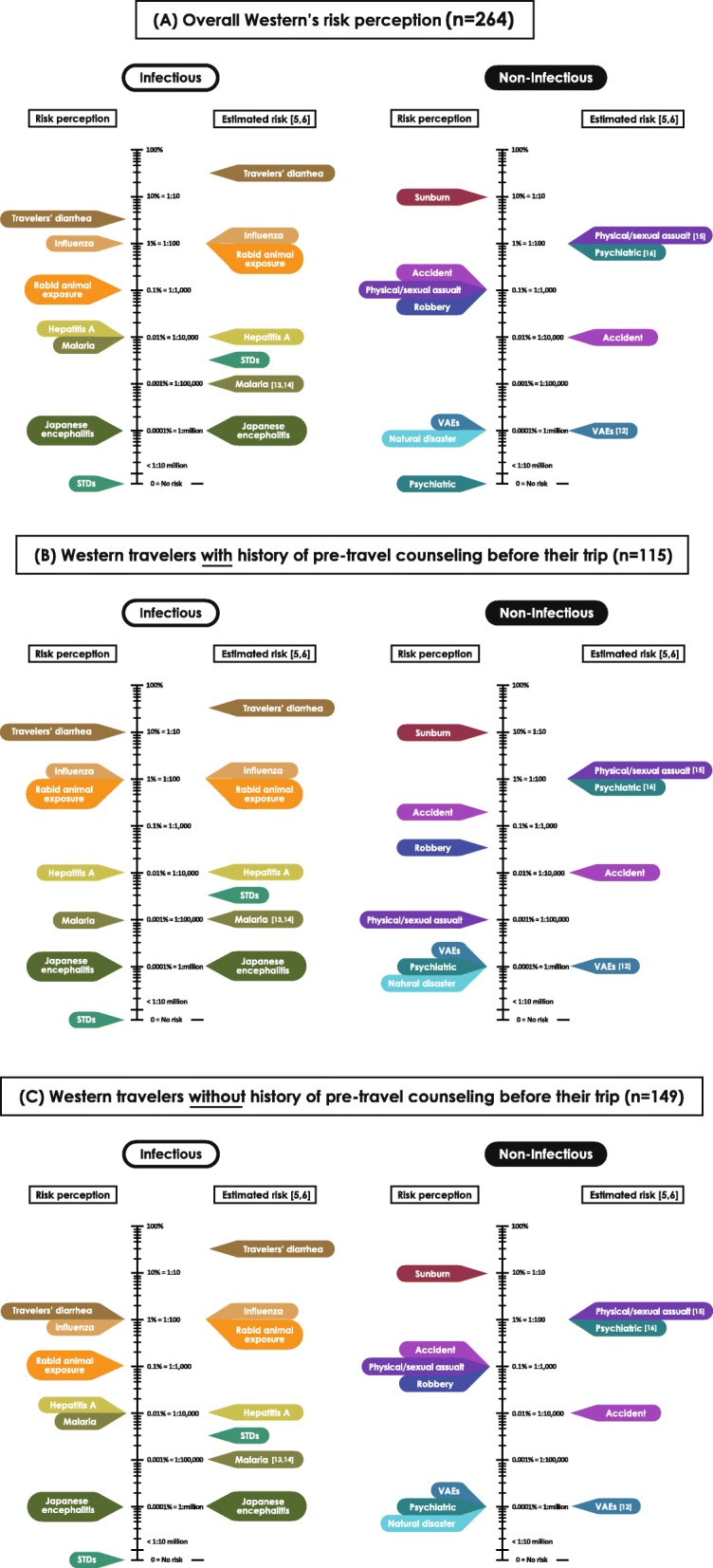
Westerners’ risk perceptions and estimated actual risk

Estimated actual risk of most vaccine-preventable health problems among western travelers has previously been described by Steffen R [5, 6]. Overall, the westerners’ perceived of risk of many health problems was accurate when compared with estimated actual risk [5, 6], e.g. for influenza, hepatitis A, Japanese encephalitis, and VAEs [12]. The risk of malaria was perceived as 0.01% (1 in 10,000) despite thorough counseling, which was an overestimation compared with actual risk of malaria infection for traveling in Southeast Asia [13, 14]. While the risk of rabid animal exposure was reported as 0.1% (1 in 1000) after counseling, it was still lower than actual risk. STDs were perceived as zero risk, regardless of counseling, in contrast with reported higher risk. Only European travelers perceived the risk of STDs compatibility with an actual risk [5, 6]. Nevertheless, the risks of malaria and rabies were correctly perceived among westerners who had received counseling before visiting the Thai Travel Clinic (*n* = 115). Surprisingly, the perceived risk for STDs remained zero regardless of pre-travel advice, like the westerners overall. A comparison of westerners’ risk perceptions and estimated actual risk for travelers’ diarrhea, physical/sexual assault [15], psychiatric problems [16], and accident are described in Fig. 5.

## Discussion

We developed a questionnaire-based pictorial scale modified from PRISM [11] to measure the risk perception of health problems among two different groups of travelers, Thais and westerners. To our knowledge, this is the first study in Thailand using a new tool with a visualized magnitude scale to measure travelers’ risk perceptions accurately. Several methodologies have been used in previous studies to measure risk perceptions among travelers, such as 3 to 5-point Likert scales and PRISM (Pictorial Representation of Illness and Self Measure) [11, 17–22], but limitations have been observed and validation remains questionable [23]. We expected that risk perceptions measured with this new measurement would yield the benefit of results directly comparable with the standard estimated risk number proposed by Steffen R [5, 6], which is generally mentioned during pre-travel advice. Moreover, it would have a benefit for lay people to understand and identify magnitude of the risk by using this tool.

The overall perceptions of travel-associated health risks of the two groups were quite different. Changed perceptions among the Thai travelers were observed for most health risks before and after pre-travel counseling, while the overall risk perceptions of the westerner group remained almost the same. Our findings indicated that different pre-travel consultation approaches are needed for Thais and western travelers [24]. Previous studies showed that culture, lifestyle and background immunity could result in different perceptions [24]. This does not mean that they do not perceive the risk. Data from our travel clinic showed that the proportion of Thai travelers visiting our travel clinic has increased [25], which could reflect increased awareness among Thai travelers. However, the common vaccine-preventable diseases recommended in the travel clinic, such as Japanese encephalitis, typhoid, hepatitis A, protect against diseases endemic in Southeast Asia [26–28]. Therefore, most Thais feel that there is no additional risk and often do not recognize the need for health advice, resulting in lower rates of Thai travelers seeking pre-travel counselling than Western travelers, especially for travel in Southeast Asia [29, 30]. In the present study, 43.6% of the westerner group received pre-travel advice before visiting our clinic. Like previous studies, they found that about 50–70% of western travelers received traveler-health information before their trips [29, 30], which can explain that their perceptions did not as much change in our study. However, the overall risk perceptions after consultations mostly agreed with the estimated actual risks of previous studies [5, 6], except for malaria, rabid animal exposure, and STDs. Interestingly, the misperceptions for malaria and rabid animal exposure were only found in the group who had not had pre-travel education.

Malaria prevention is an important health issue addressed during pre-travel consultations in most travel clinics, especially in western countries [31]. The western travelers perceived that risk of malaria in Southeast Asia was high when compared with estimated actual risk [13, 14]. Interestingly, travelers who received pre-travel consultations had accurate perceptions of malaria risk when compared with actual risk. The standard recommendations from the CDC or UK guidelines stress that malaria risk is essential pre-travel health information [32, 33]. Therefore, most western travelers who sought pre-travel health information were aware of the risk of malaria and knew it to be a serious, potentially fatal, disease [20]. However, the risk of malaria in moderate to low risk areas was well described as 1 in 10,000 [34]. Travelers would only need malaria chemoprophylaxis for security reasons [35]; some believed malaria could be 100% prevented by chemoprophylaxis [20]. This misperception might result in the lack of other preventive measures to prevent infection, such as avoiding mosquito bites. We would highlight the essential of health education to travel medicine practitioners for including other malaria prevention measures such as mosquito repellent use and long sleeve clothes in their pre-travel health counseling together with an advice for chemoprophylaxis.

The western travelers had increased awareness of the risk of rabid animal exposure after the pre-travel health consultation. However, the overall risk perception of rabid animal exposure remained lower than the estimated actual risk [36]. Travelers who previously received pre-travel counseling had accurate risk perceptions. A study among Western backpackers visiting Thailand also found that, despite 23.6% previously undergoing counseling, many were still unaware of and unprepared for the risk of rabies [37]. However, while they sought health information before their trip, 73.1% did not have rabies pre-exposure vaccination(s) [38]. Apparently, over half of rabid animal exposure among travelers has occurred in Asia [39] and the incidence of potential rabid animal exposure was reported as 56 per month per 1000 short-term western travelers visiting Southeast Asia [40]. Therefore, the risk of rabid animal exposure should be emphasized and encouraged on pre-exposure immunization during pre-travel advice before travel to Southeast Asian countries [38].

Risk perception of sexually transmitted diseases was rated as zero among most western travelers, regardless of counseling. We assumed that it might have been neglected by traveler-health care providers during their consultations. A previous study demonstrated a similar finding, that travelers underestimated the risk of STDs [11] because they did not plan to have sex during their trips. However, most sexual behavior occurred unintentionally [41]. The risk of sexually transmitted diseases has been addressed in previous studies [42]. One-fourth of backpackers had casual sex during their trips [43] and 36.8% of these reported inconsistent/no condom use. Thus, awareness of the risk of STDs among travelers should be a priority for both travelers and travel medicine practitioners during pre-travel communication.

### Limitation

The potential for selection bias among the study group is acknowledged, because the study populations were enrolled at the Thai Travel Clinic and might already possess greater risk awareness than the general traveler population. Further study is recommended with different study populations (in addition to hospital-based), travel destinations in other tropical and sub-tropical regions, and with larger sample sizes.

## Conclusion

An overall change in risk perceptions was noted among the Thai travelers, whereas the perceived risk levels of the westerners only changed slightly, and largely agreed with the estimated risk numbers for most travel-related health risks; especially among the group that previously received advice in their home countries before traveling. Differences in risk perceptions were evident between the two groups. Therefore, this highlight the importance of obtaining pre-travel advice in one’s home country before travelling. Raised awareness of the risks should be emphasized during consultations for underestimated health risks, especially for rabid animal exposure and sexually transmitted diseases. Our study showed the benefit of the novel risk perception-pictorial scale in the ability of directly comparison with an actual risk. Further study evaluating perception using this novel tool is encouraged to specify validation and generalization. Moreover, study on the estimated risks for Thai travelers to developing countries is also needed.

## Supplementary information

**Additional file 1.** The funnel plots illustrated the distribution of answer in 14 health problems on pre- and post-travel counseling among both groups of the participants.

**Additional file 2.** Subgroup analysis of risk perception pre- and post-travel counseling differentiated by nationality of western travelers toward 14 health problems.

## Data Availability

The raw data and the questionnaire used in this study are available at the Faculty of Tropical Medicine, Mahidol University. The corresponding author can be contacted via email: wasin.mat@mahidol.edu

## Abbreviations

STD: Sexually Transmitted Diseases
VAEs: Vaccine Adverse Events
