# Supplementary material for: Risk perception of health problems among travelers visiting a travel clinic in Bangkok, Thailand
Source: Trop Dis Travel Med Vaccines. 2020 May 20;6:7. doi: 10.1186/s40794-020-00108-0 (PMC7238588; doi:10.1186/s40794-020-00108-0)

Additional file 1 The funnel plots illustrated the distribution of answer in 14 health problems on pre- and post-travel counseling among both groups of the participants

The distribution of perception among Thai and western travelers were presented for all 14 travel-related health risks comparing pre- and post-consultation.

Thai travelers

Western travelers

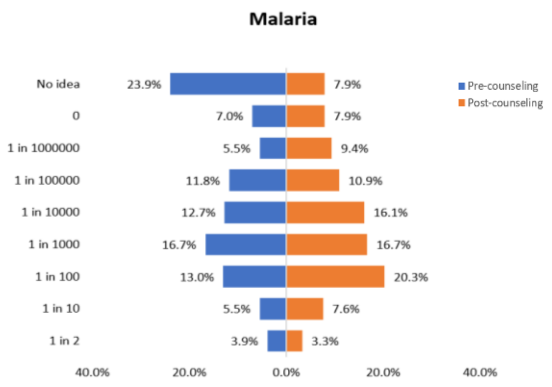

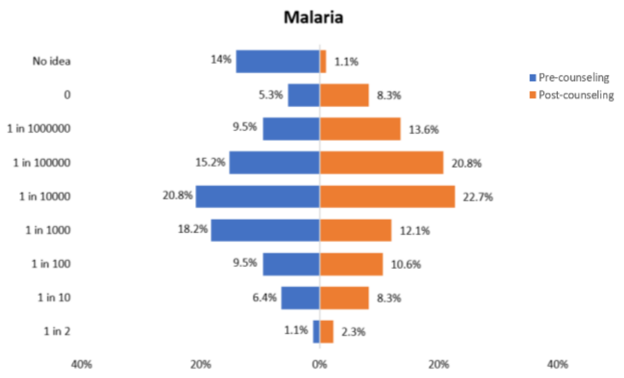

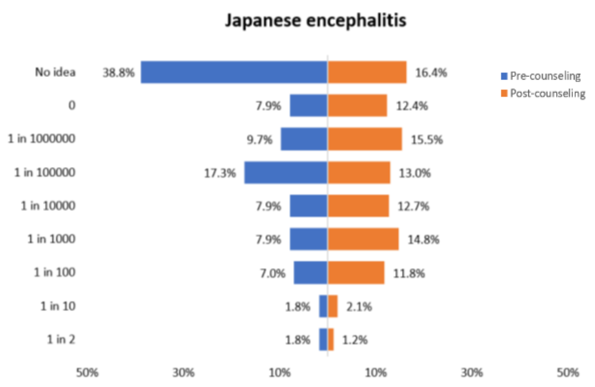

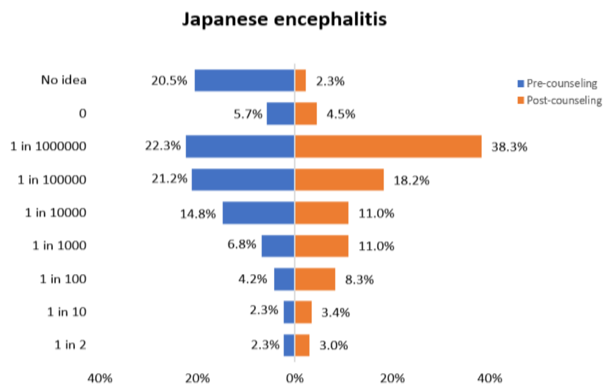

Thai travelers

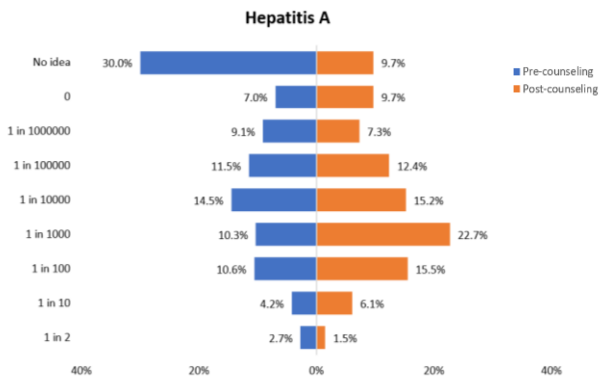

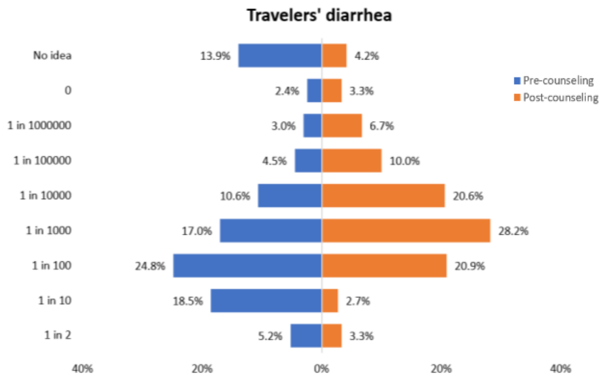

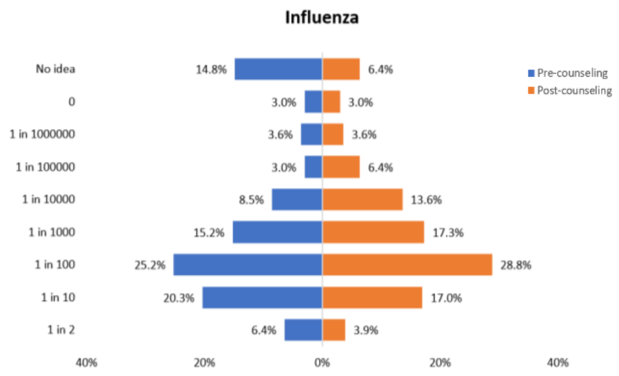

Western travelers

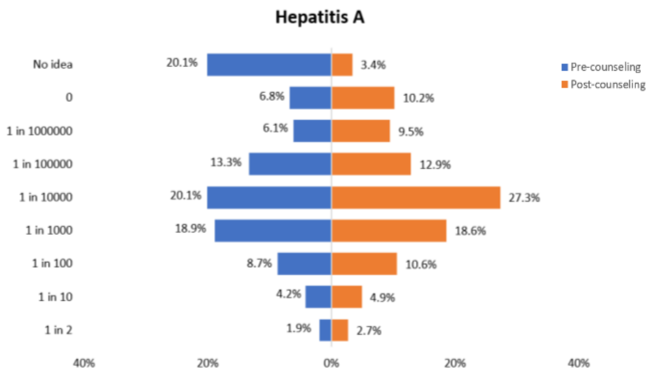

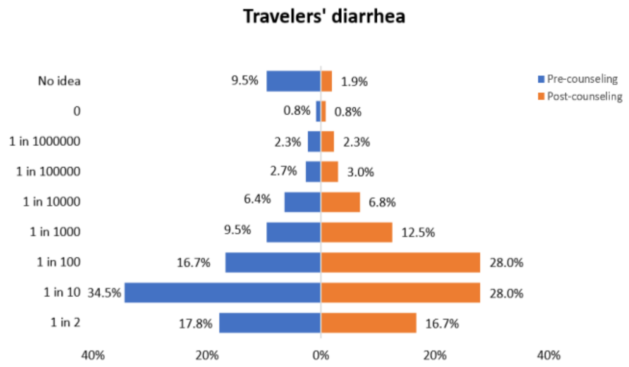

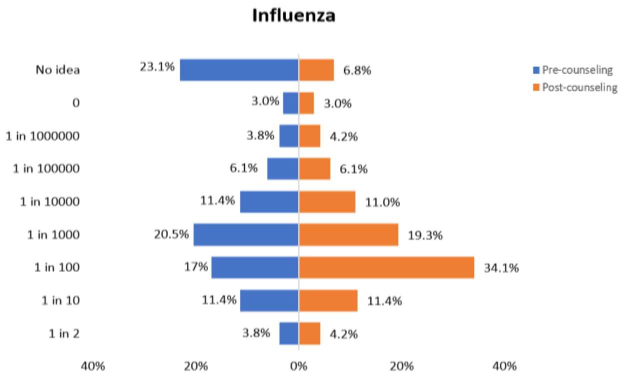

Thai travelers

Western travelers

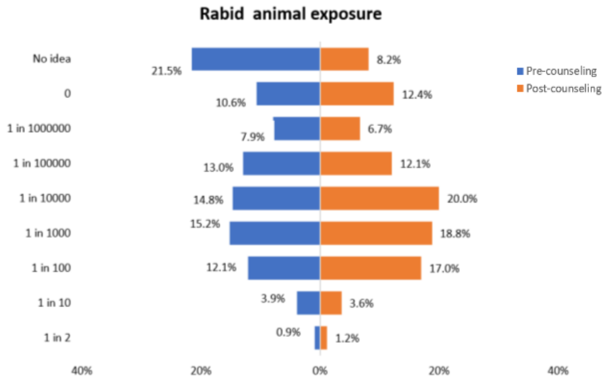

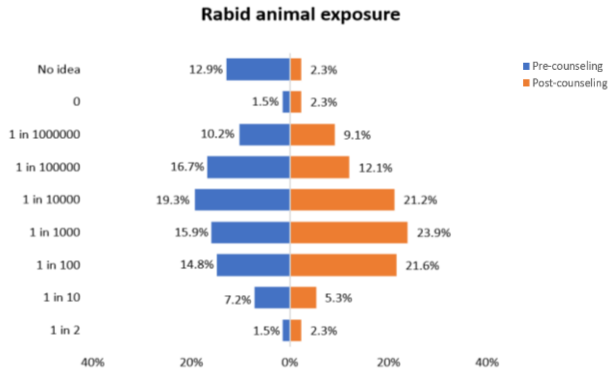

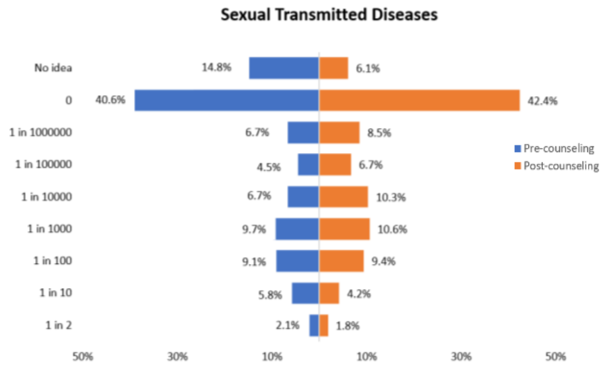

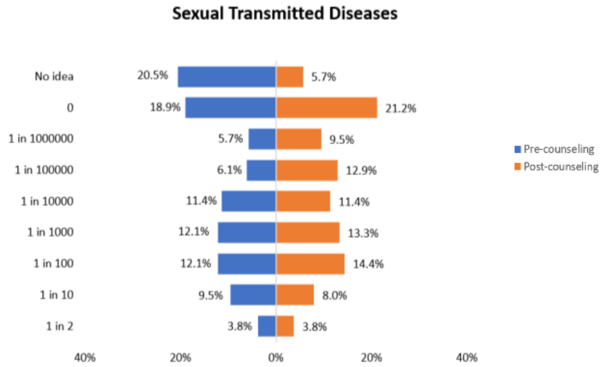

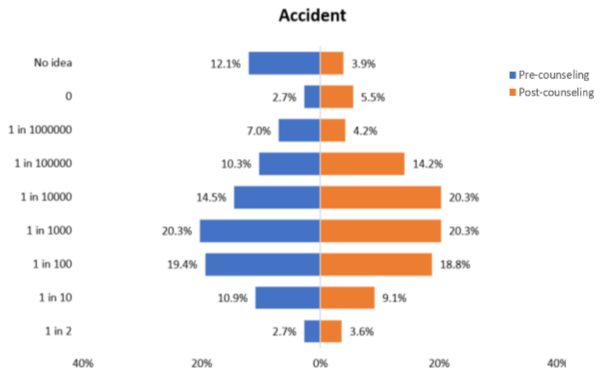

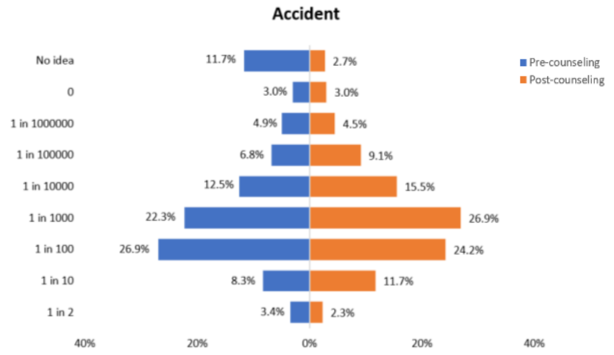

Thai travelers

Western travelers

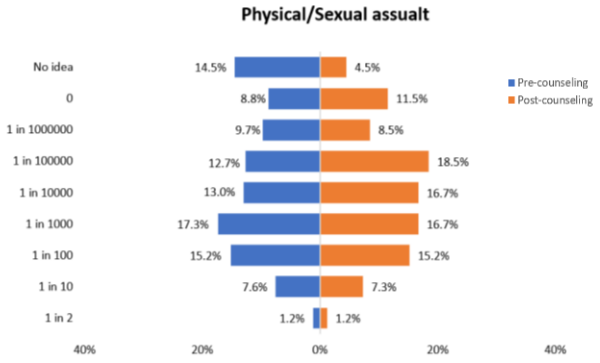

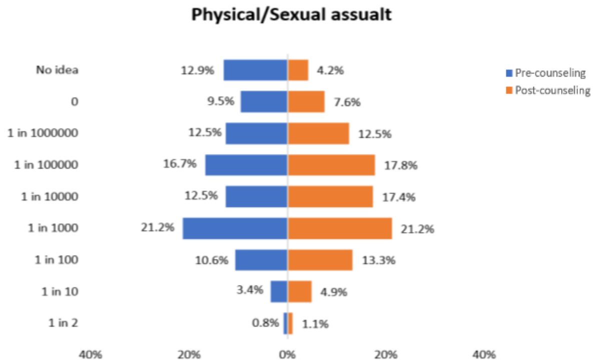

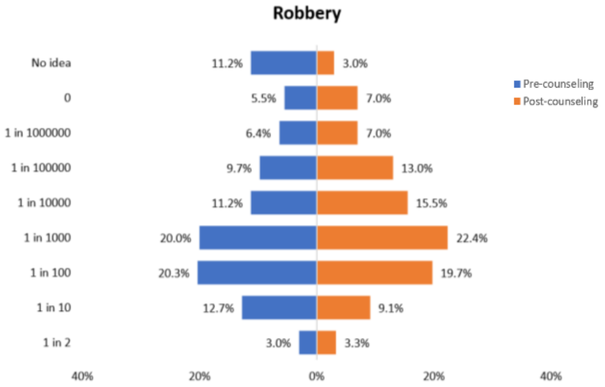

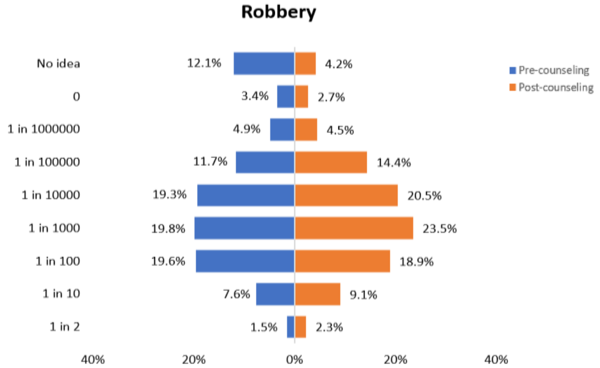

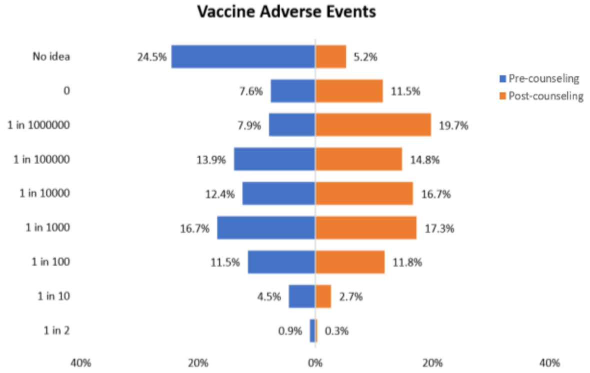

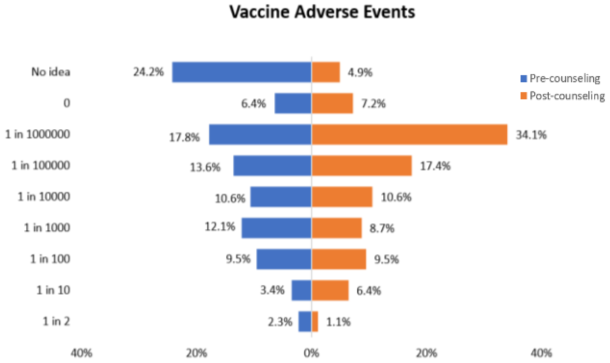

Thai travelers

Western travelers

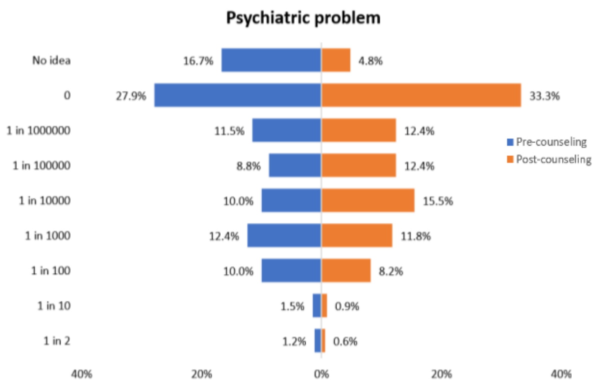

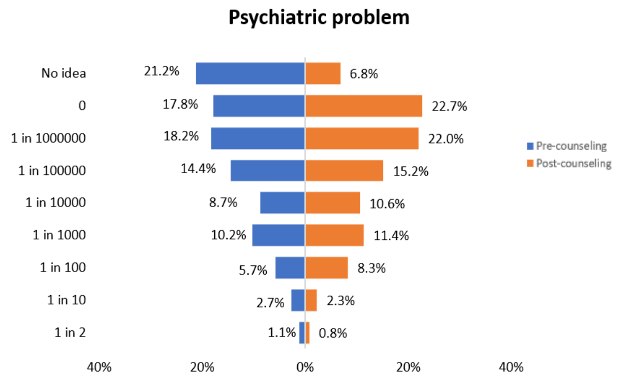

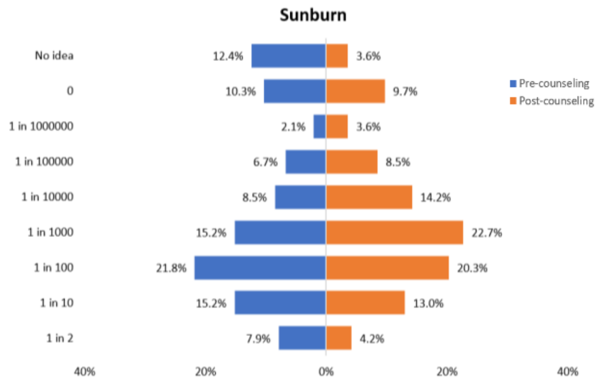

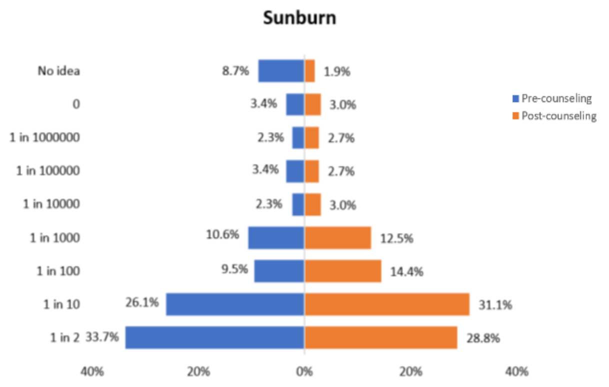

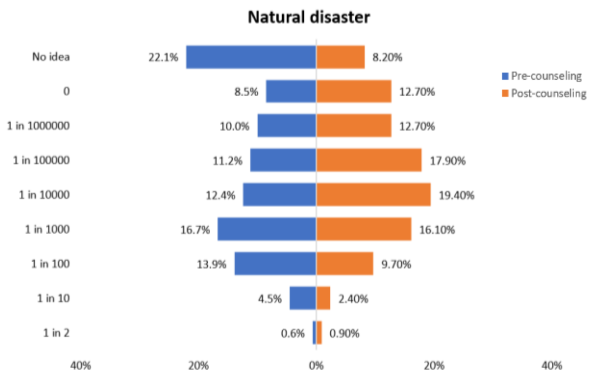

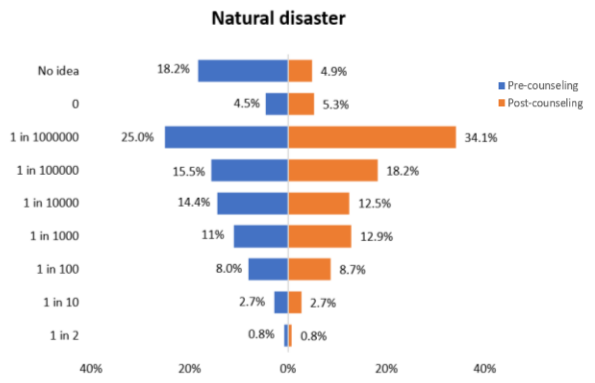

Supplement: Supplementary file 1 — Additional file 1. The funnel plots illustrated the distribution of answer in 14 health problems on pre- and post-travel counseling among both groups of the participants. [file 40794_2020_108_MOESM1_ESM.pdf]
