# Supplementary material for: Risk perception of health problems among travelers visiting a travel clinic in Bangkok, Thailand
Source: Trop Dis Travel Med Vaccines. 2020 May 20;6:7. doi: 10.1186/s40794-020-00108-0 (PMC7238588; doi:10.1186/s40794-020-00108-0)
Supplement: Supplementary file 2 — Additional file 2. Subgroup analysis of risk perception pre- and post-travel counseling differentiated by nationality of western travelers toward 14 health problems. [file 40794_2020_108_MOESM2_ESM.pdf]

Additional file 2 Subgroup analysis of risk perception pre- and post-travel counseling differentiated by nationality of western travelers toward 14 health problems

These charts demonstrated mode of participant's response for the magnitude of risk in each health problem comparing pre- and post-travel consultation. The charts illustrated below were categorized by nationality of the participants: Europe, North America, and Australia/New Zealand.

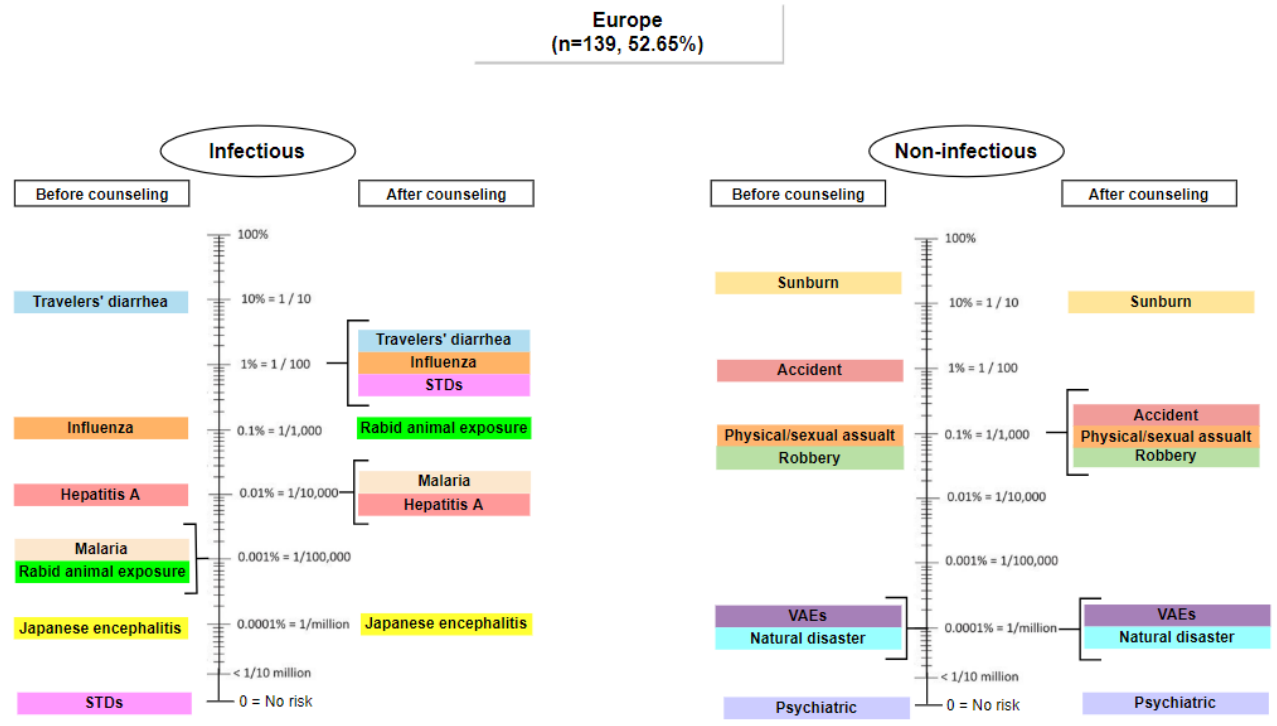

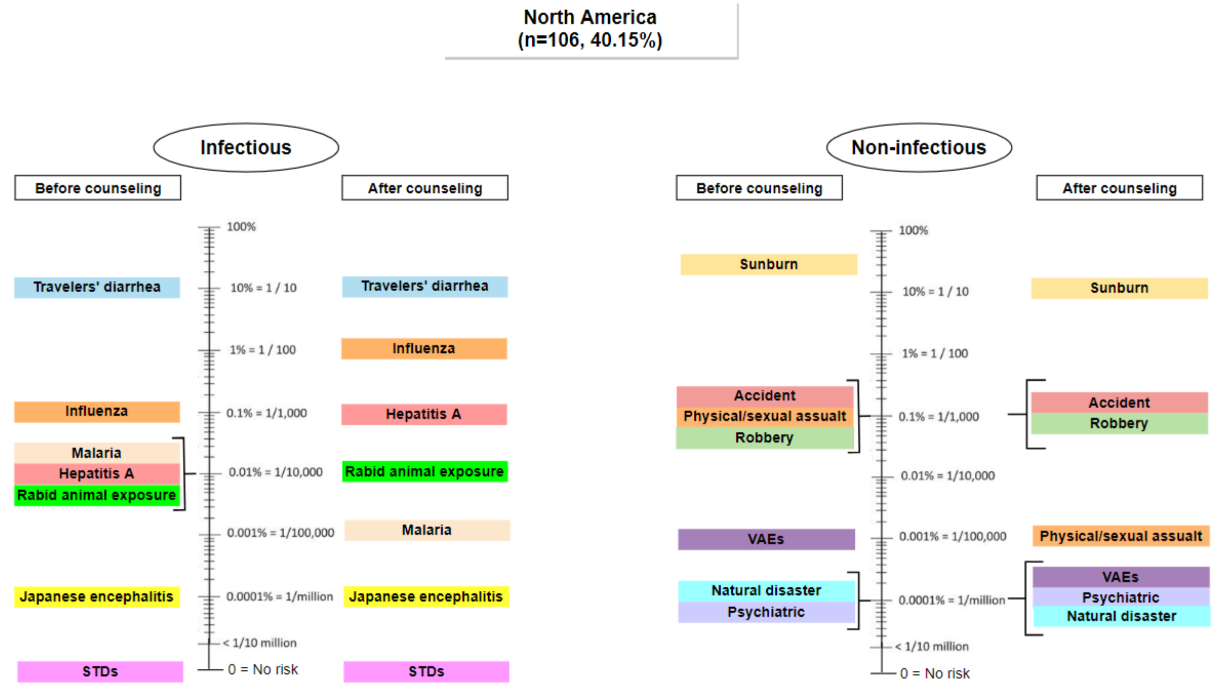
North America
(n=106, 40.15%)
Infectious
Before counseling
After counseling
100%
10% = 1 / 10
1% = 1 / 100
0.1% = 1/1,000
0.01% = 1/10,000
0.001% = 1/100,000
0.0001% = 1/million
< 1/10 million
0 = No risk
Travelers' diarrhea
Influenza
Malaria
Hepatitis A
Rabid animal exposure
Japanese encephalitis
STDs
Travelers' diarrhea
Influenza
Hepatitis A
Rabid animal exposure
Malaria
Japanese encephalitis
STDs
Non-infectious
Before counseling
After counseling
Sunburn
Accident
Physical/sexual assualt
Robbery
VAEs
Natural disaster
Psychiatric
Sunburn
Accident
Robbery
Physical/sexual assualt
VAEs
Psychiatric
Natural disaster

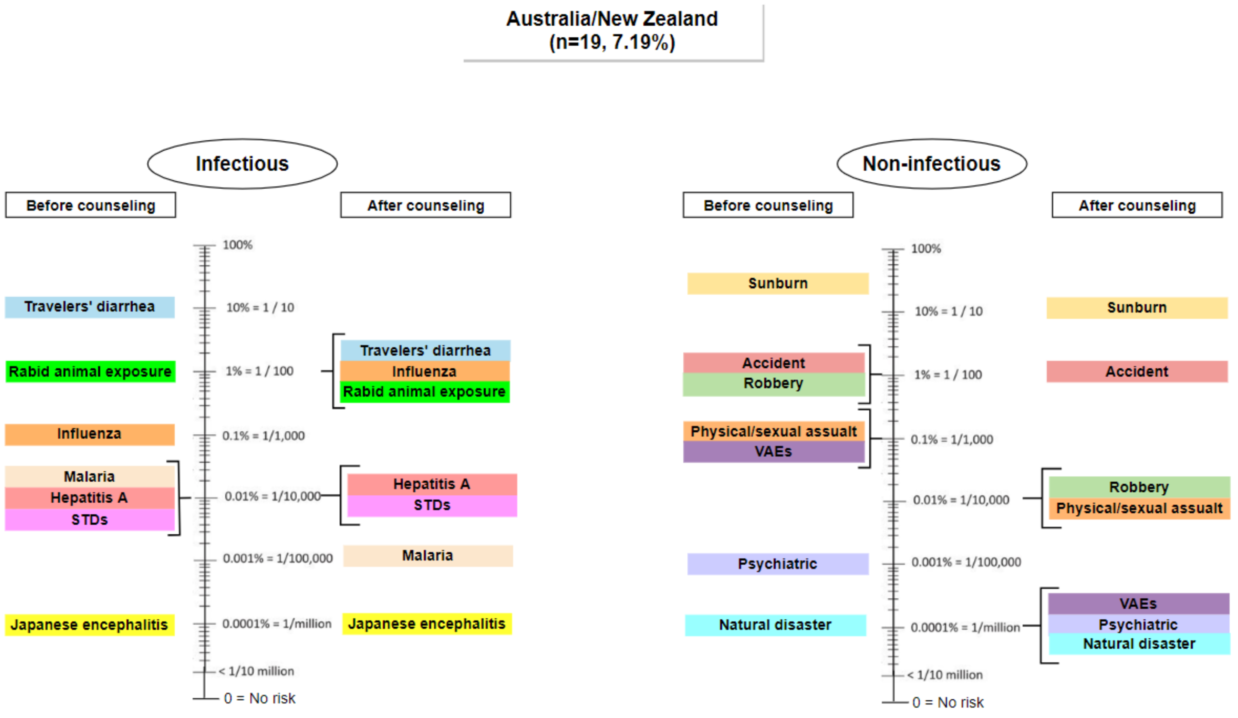
Australia/New Zealand
(n=19, 7.19%)
Infectious
Before counseling
After counseling
100%
10% = 1 / 10
1% = 1 / 100
0.1% = 1/1,000
0.01% = 1/10,000
0.001% = 1/100,000
0.0001% = 1/million
< 1/10 million
0 = No risk
Travelers' diarrhea
Rabid animal exposure
Influenza
Malaria
Hepatitis A
STDs
Japanese encephalitis
Travelers' diarrhea
Influenza
Rabid animal exposure
Hepatitis A
STDs
Malaria
Japanese encephalitis
Non-infectious
Before counseling
After counseling
Sunburn
Accident
Robbery
Physical/sexual assualt
VAEs
Psychiatric
Natural disaster
Sunburn
Accident
Robbery
Physical/sexual assualt
VAEs
Psychiatric
Natural disaster
